# Phyllotaxis without symmetry: what can we learn from flower heads?

**DOI:** 10.1093/jxb/erac101

**Published:** 2022-03-11

**Authors:** Przemyslaw Prusinkiewicz, Teng Zhang, Andrew Owens, Mikolaj Cieslak, Paula Elomaa

**Affiliations:** Department of Computer Science, University of Calgary, Calgary AB T2N 1N4, Canada; Department of Agricultural Sciences, Viikki Plant Science Centre, University of Helsinki, 00014 Helsinki, Finland; Department of Computer Science, University of Calgary, Calgary AB T2N 1N4, Canada; Department of Computer Science, University of Calgary, Calgary AB T2N 1N4, Canada; Department of Agricultural Sciences, Viikki Plant Science Centre, University of Helsinki, 00014 Helsinki, Finland; University College Dublin, Ireland

**Keywords:** Asteraceae, *CLAVATA*, divergence angle, fasciation, Fibonacci number, flower head, Hofmeister hypothesis, modeling, parastichy, phyllotaxis

## Abstract

Phyllotaxis is commonly considered in the context of circular meristems or receptacles, yet non-circular (fasciated) structures also give rise to new primordia and organs. Here we investigate phyllotactic patterns in fasciated flower heads in the Asteraceae plant family. We begin by surveying the phenomenon of fasciation. We then show that phyllotactic patterns in fasciated heads can be generated by removing the inessential assumption of circularity from the previously published model of gerbera heads. To characterize these patterns, we revisit the conceptual framework in which phyllotactic patterns are commonly described. We note that some notions, in particular parastichies and parastichy numbers, maintain their significance in non-circular phyllotaxis, whereas others, in particular the divergence angle, need to be extended or lose their role. These observations highlight a number of open problems related to phyllotaxis in general, which may be elucidated by studies of fasciated heads.

## Teaser

A survey and model of fasciated flower heads separate fundamental and derived characteristics of phyllotactic patterns and highlight open problems in phyllotaxis research.

## Introduction

**Fig. 1.**
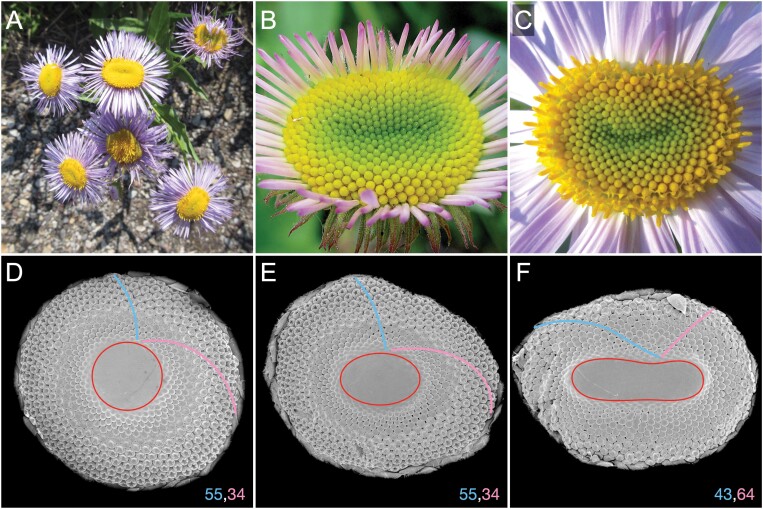
Examples of phyllotaxis in naturally occurring fasciated heads. (A) Photographs of a fleabane plant (*Erigeron* sp.) with multiple fasciated heads (Waterton National Park, Canada, July 2021). Fasciated heads in fleabane are frequent and easy to spot. (B, C) Macro photographs of fasciated fleabane heads showing the arrangement of their florets. (D–F) Scanning electron microphotographs of patterns developing in circular (D) and elliptic (E, F) gerbera heads. Fibonacci parastichy numbers characteristic of circular gerbera heads (D) are preserved in the elliptic head (E), but not preserved in the more elongated head (F). The sample fixation, critical point drying, and SEM analysis were performed as described by Zhang *et al.* (2017). Photograph (C) by Lynn Mercer, used with permission.

Phyllotaxis—the arrangement of plant organs—has attracted the interest of scientists for centuries (Schwabe, 1984; Adler *et al.*, 1997; Barabé and Lacroix, 2020). To date, the most extensive studies have been carried out on the model plants Arabidopsis and tomato (Kuhlemeier, 2017), in which the initiation of organ primordia can be idealized as a process taking place at a radially symmetric shoot apical meristem (SAM) at regular time intervals, with the divergence angle between consecutive primordia close to 137.5° (Mündermann *et al.*, 2005). In nature, however, phyllotactic patterns also arise in the absence of radial (or any other) symmetry and rhythmic production of primordia (Fig. 1). This scenario has received much less attention; consequently, the conceptual framework needed to describe phyllotaxis in the absence of radial symmetry is not well developed. Here we survey selected results and present a hypothetical model of phyllotactic patterns on fasciated meristems. In addition to being an interesting object of study themselves, these patterns provide an insight into the general mechanisms of phyllotaxis, and the application range of different parameters used to characterize them.

Our stepping stone is the recently developed model of phyllotaxis in the heads of *Gerbera hybrida*, a member of the large Asteraceae family of plants (Zhang *et al.*, 2021). As in other Asteraceae species, florets in gerbera heads are typically arranged into Fibonacci numbers of conspicuous spirals (parastichies): 34 running in one direction and 55 running in the opposite direction (Fig. 1D, E). As shown by van Iterson (1907) and subsequently explored by Douady and Couder (1996), among others, in some plants such an arrangement may result from a continuous elaboration of the pattern from cotyledons to leaves on the stem to involucral bracts and florets in the head. However, in gerbera heads, spiral phyllotactic patterns with high Fibonacci parastichy numbers emerge *de novo*. The nature of the process resulting in such emergence has long been an open question (Green *et al.*, 2008). Based on visualizations of auxin activity in transgenic gerbera lines, Zhang *et al.* (2021) proposed a model that provides an explanation. Although focused on circular heads, this model has also shown that transient departures of the patterning process from circular symmetry do not necessarily disturb the final pattern. This intriguing result, consistent with previous experimental data (Szymanowska-Pułka, 1994), has led us to the exploration of phyllotaxis in fasciated heads presented here. After a short overview of fasciation, we extend the gerbera model to fasciated heads and, in this context, we discuss how patterns in fasciated heads may contribute to the general understanding and characterization of phyllotaxis.

## Phyllotaxis in fasciated meristems

Fasciation refers to shape changes in the shoot apex ranging from relatively mild distortions, such as elliptic rather than circular shapes, to drastic deformations such as strap- or ribbon-like shapes (Worsdell, 1905; White, 1948). These changes typically include enlargement or deformation of the meristem, which may affect organ arrangement and organ numbers, as well as widening and flattening of organs such as the floral stem. The resulting structures are commonly observed in distinct plant species across the plant kingdom and have traditionally been described as monstrosities or botanical curiosities (Masters, 1869; Schoute, 1936; White, 1948; Choob and Sinyushin, 2012). Phyllotactic patterning in fasciated meristems has not been extensively studied; below, we review selected results.

It is well established that the zonal organization of the SAM is regulated by the WUSCHEL (WUS)/CLAVATA3 (CLV3) feedback loop, which maintains the size of the stem cell niche and sets the context for robust organ initiation in the *Arabidopsis thaliana* model plant (reviewed by Gaillochet *et al.*, 2015; Somssich *et al.*, 2016; Fuchs and Lohmann, 2020). In various plant species, meristem enlargement is typically associated with genetic defects in this regulatory loop, CLV3 peptide perception or signaling, or CLV3-independent upstream regulators of *WUS* (reviewed by Soyars *et al.*, 2016; Kitagawa and Jackson, 2019). In Arabidopsis, the mutants *clv1*, *clv2*, and *clv3* all develop enlarged meristems due to the expanded *WUS* expression domain (Leyser and Furner, 1992; Fletcher *et al.*, 1999; Schoof *et al.*, 2000). The increase in meristem size begins in the embryo and continues in both vegetative and floral apices. Eventually, the inflorescence meristems in the strong *clv1* and *clv3* mutants grow as a ring, as a linear, strap-like structure, or as a large mass (Clark *et al.*, 1993; Fletcher *et al.*, 1999). In *clv1* mutants, altered divergence angles lead to changes in the phyllotaxis of leaves (Leyser and Furner, 1992), and the spiral arrangement of flowers is lost due to the emergence of larger numbers of flower primordia at the apical meristems (Clark *et al.*, 1993). In meristems with linear fasciation, numerous flower primordia form along the meristematic line (Clark *et al.*, 1993). Likewise, in *clv3* mutants, the primordia formed at the SAM periphery do not follow a recognizable phyllotactic pattern, although at a local scale the positions of new primordia are predictable: they emerge ‘in a wedge-shaped portion of the SAM periphery, between two older primordia that are still in direct contact with the SAM through the already formed axils, and whose developmental stages are preceding the sepal formation.’ (Szczęsny *et al*., 2009, p. 683). This observation is consistent with our hypothesis that phyllotactic patterns in asymmetric heads result from the normal local patterning mechanism operating in an unusual global context.

Apart from these often severe genetic effects, meristem shape can be affected by developmental constraints. For instance, the inflorescences in *Drimys winteri* (Winteraceae), a shrub representing a basal lineage of angiosperms, are racemes that develop elliptic lateral meristems, whereas the terminal meristems are larger and circular (Doust, 2001). The round terminal meristems initiate organs uniformly, with a constant divergence angle, in either a spiral or a whorled pattern. The elliptic meristems show greater diversity in phyllotactic patterns and initiate organs non-uniformly: first at the meristem poles, and only later along the sides. Doust (2001) proposed that this pattern could result from physical pressure, which inhibits organ initiation on the sides of the elliptic meristem. Alternatively, primordia could preferentially initiate at the poles because they are more distant from the meristem center, compared with the meristem sides.

Our recent data focus on understanding phyllotactic patterning in Asteraceae flower heads with high spiral numbers of florets (Zhang *et al.*, 2021). The Asteraceae species may develop fasciated heads ranging from oval-shaped (Fig. 1B) to more severe ribbon-like phenotypes (Pavlovic *et al.*, 2013). Phyllotactic patterns of flower heads have most extensively been recorded by Szymanowska-Pułka (1994), who examined over 1000 mature flower heads of *Carlina acaulis* L. and showed that most heads (93%) followed highly regular phyllotaxis with the main Fibonacci series. Notably, in her datasets, the floret spirals in the oval-shaped, fasciated flower heads still followed either exact Fibonacci numbers (cf. fig. 19) or multiples thereof in multijugate patterns (cf. figs 9a, 10a), indicating that departures from the circular head shape need not cause deviation in parastichy numbers. Outside Asteraceae, regular Fibonacci numbers of spirals were also detected in the strongly fasciated meristems of romanesco curds (cf. fig. 9D in Kieffer *et al.*, 1998).

The genes that cause head fasciation are not known. Interestingly, as the gerbera head develops, the *GhCLV3* expression domain expands, delineating the undifferentiated meristematic cells and the position of the active ring where the floret patterning occurs (Zhang *et al.*, 2021). In contrast to Arabidopsis, the expanded *CLV3* expression in gerbera does not result in uncontrolled cell proliferation, fasciation, or abnormalities in phyllotactic pattern, which indicates that the cell division regulation is not disrupted with respect to wild-type heads. In sunflower, a spontaneous mutant, *stem fasciated* (*stf*), was shown to be under the control of a single recessive gene, the identity of which is not known (Fambrini *et al.*, 2006). The mutant plant developed an enlarged vegetative SAM leading to flat stems, whorled leaf arrangement, and enlarged head meristems, with extra apical domes on the meristem surfaces and multiple inflorescences (Fambrini *et al.*, 2006). The usual phyllotactic pattern of heads with spiral numbers of 34 and 55 was lost. Interestingly, the individual florets developed normally, indicating that the mutation does not affect flower meristems.

In single flowers, analogous structures with linear fasciation can be found in association with highly increased organ numbers (polymery) (Endress, 2014). An extreme case is the ornamental plant *Tupidanthus calyptratus* (Araliaceae), which develops a butterfly-shaped flower meristem and an extremely high number (>100) of stamens and carpels arranged in highly meandering single whorls (Sokoloff *et al.*, 2007). The numerous stamens develop simultaneously along the irregular meristematic rim, and the carpels form in alternating positions with the stamens. The flowers do not show radial symmetry at any stage of development. A related species, *Schefflera subintegra*, shows less extreme polymery, but still has large, elliptic flowers with tens of stamens and carpels in single whorls (Nuraliev *et al.*, 2014). In mature flowers, the carpels are arranged in parallel rows facing each other. Molecular studies are needed to verify whether these phenotypes result from mutation(s) of the *CLV* gene(s) or modifications in other genes (e.g., those upstream of *CLV3* or those targeting *WUS* independently of *CLV*), and to explore the underlying evolutionary trajectories behind these forms. An interesting question is whether these polymeric forms are maintained in nature because they possess some functional or ecological advantage.

## The gerbera model of spiral phyllotaxis

The gerbera model (Zhang *et al.*, 2021) was originally implemented on circular heads. Nevertheless, it does not fundamentally depend on the assumption of circularity; consequently, here we extend it to fasciated heads. The model is based on the Hofmeister (1868)/Snow and Snow (1931, 1952) hypothesis, according to which new primordia emerge in the organogenetic zone—referred to as the active ring—where and when there is enough space for them. This process is controlled by the interplay between the growth of the head meristem (receptacle) and the changing position of the active ring over time. The dynamics of this interplay divide phyllotactic patterning in gerbera heads into three phases.

**Fig. 2.**
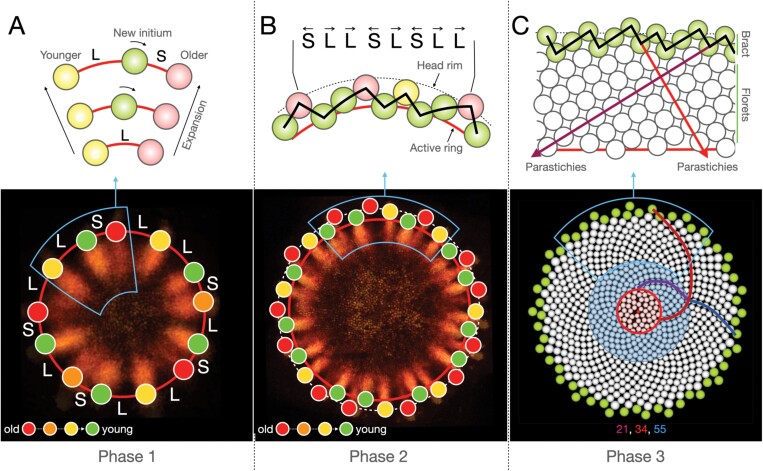
The gerbera phyllotaxis model of Zhang *et al.* (2021). Auxin patterning was visualized in transgenic gerbera plants expressing the *DR5rev::3XVENUS-N7* auxin reporter. (A) In Phase 1, the incipient primordia are inserted on the head rim between previously initiated primordia and tend towards their older neighbors, creating a pattern of long (L) and short (S) intervals. The resulting asymmetry leads to the progression of primordia numbers according to the Fibonacci sequence. (B) The active ring (red line) separates from the head rim (dashed line), resulting in the formation of a zig-zag pattern front. The numbers of ‘zigs’ and ‘zags’ are consecutive Fibonacci numbers. (C) The active ring propagates to the head center, extending the zig-zag pattern in (B) to a typical spiral phyllotactic pattern with intersecting parastichies. The number of parastichies decreases at the outer boundary of the blue circle. The pattern becomes chaotic at the outer boundary of the red circle.

Phase 1 (Fig. 2A) is essentially a one-dimensional process taking place on the active ring coinciding with the rim of a growing head. The first primordium is assumed to emerge in an arbitrary position on the rim. The subsequent rim expansion creates space into which new primordia are inserted. As the expansion continues, these primordia are displaced laterally, tending towards their older neighbors (Primordium 2 is a special case, with the direction of displacement chosen arbitrarily). As a result, the space between primordia is partitioned unevenly. For a range of displacement rates, this asymmetry leads to the progression of primordia numbers according to the Fibonacci sequence: 1, 2, 3, 5, 8, 13, ….

In Phase 2 (Fig. 2B), the pattern gradually acquires a two-dimensional character. While the head continues to grow, the active ring begins to dissociate itself from the rim. As a result, new primordia emerge closer to the head center than their older neighbors, creating a zig-zag pattern of ascending and descending segments of the pattern front. Elaborating upon the initial one-dimensional pattern, these oppositely oriented segments often appear in consecutive Fibonacci numbers.

Phase 3 (Fig. 2C) has a decidedly two-dimensional character. The space for the new primordia is created by the active ring that continues to propagate towards the head center, thus moving away from the primordia created earlier. The addition of new primordia elaborates the initial zig-zag pattern into a pattern of parastichies. With the ring shrinking, the number of parastichies decreases in jumps following the reversed Fibonacci sequence. Eventually, patterning is disrupted by the proximity of primordia formed at different points of the highly contracted ring, and the pattern becomes chaotic.

## Extending the gerbera model to fasciated heads

The adaptation of the above model to fasciated heads requires addressing two questions. (i) How can the growth of the head, and the propagation of the active ring, be described in the non-circular case? (ii) What distance measure is suitable to evaluate the availability postulated by the Hofmeister/Snow and Snow hypothesis in the absence of circularity?

The growth of shoot apical meristems has already been analyzed in selected plants (e.g., Dumais and Kwiatkowska, 2002; Kwiatkowska, 2006; Kierzkowski *et al.*, 2012), including several Asteraceae species (Hernandez 1995; Dosio *et al.*, 2006; Zhang *et al.*, 2021). Unfortunately, data pertinent to fasciated heads are difficult to obtain due to the relatively lower frequency of their occurrence and less stereotypical development. Consequently, here we model phyllotaxis on fasciated heads using two simplifying assumptions: that the receptacle is planar (flat) and that, in Phase 3, the growth of the head is negligible, with patterning driven by the propagation of the active ring. These assumptions focus the model on the curves representing the rim and the active ring.

**Fig. 3.**
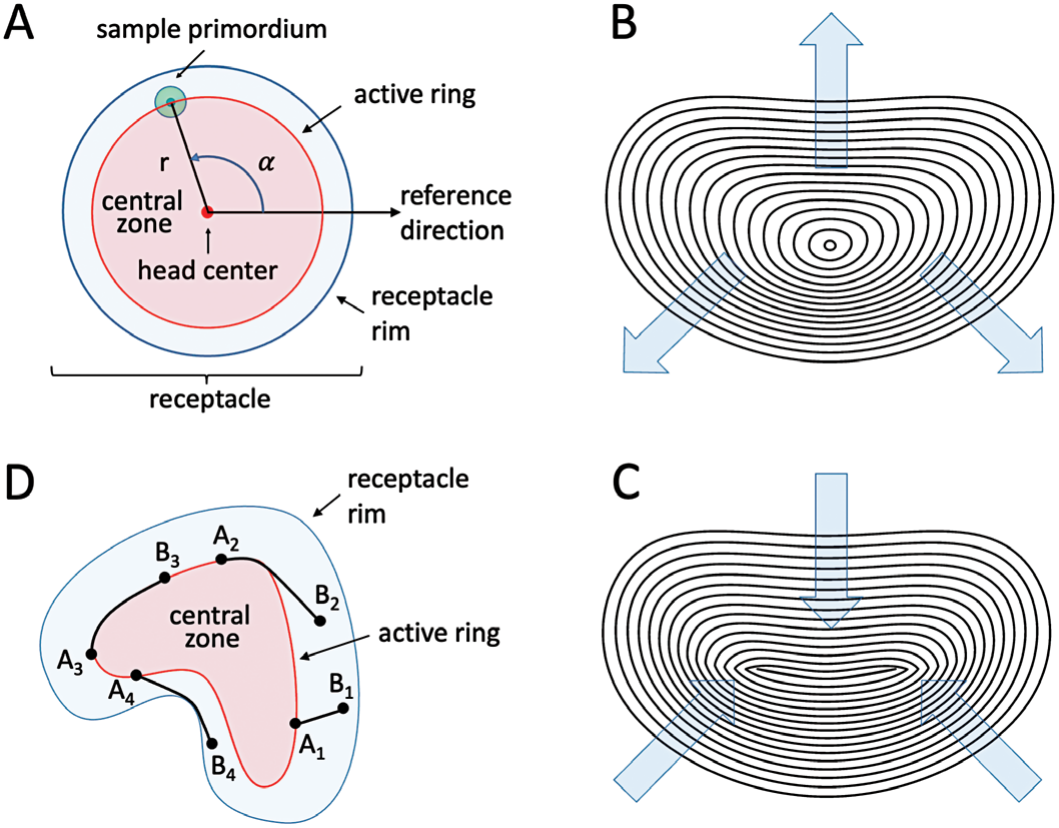
Extending the gerbera model to fasciated heads. (A) The specification of primordia positions in polar coordinates, commonly used for circular heads. A primordium is described in terms of its distance from the head center and the angle with respect to a chosen reference direction. Propagation of the head rim and the active ring can be described as changes in their radii. (B, C) Example of active ring expansion (B) and contraction (C) specified using keyframing. (D) Illustration of the notion of the shortest path between points *A*_i_ (lying on the active ring) and points *B*_i_, confined to the space between the active ring and the receptacle rim. The length of path *A*_1_*B*_1_ is the straight-line distance between its endpoints, the length of path *A*_3_*B*_3_ is the arc-length distance between its endpoints (measured along the curve), and the lengths of paths *A*_2_*B*_2_ and *A*_4_*B*_4_ are combinations of arc-length and straight-line distances.

In planar models of circularly symmetric phyllotaxis, all elements of the pattern are typically described using polar coordinates—in terms of distances from the head center and angles from a reference direction (Fig. 3A). For fasciated heads, this approach is impractical, because, in general, the head ‘center’ is no longer well defined. However, the curves representing the rim or the active ring may themselves serve as a reference. The development of these curves can then be described in terms of the propagation of each curve point in the normal directions, namely perpendicular to the (tangent to the) curve at that point (Sethian, 1999). This description reduces to the usual definition of growth in terms of changes to the radius in the circular case, but extends to any smooth curve. Another possibility, referred to as keyframe animation (Burtnyk and Wein, 1971; Mosca *et al.*, 2018) and employed in our simulations, is the interpolation between globally defined curves representing snapshots of the rim or active ring positions over time (Fig. 3B, C). This method is particularly useful in descriptive models of head development, where the key frames represent empirical data. Different variants and combinations of both techniques are also possible.

The growth of circularly symmetric heads is commonly assumed to be rotationally invariant, which means that it is the same at each point of the rim. For fasciated heads, however, a more specific characterization of the rim expansion is needed. A suitable notion is intrinsic growth, quantified as the elongation of unit curve segments over time [relative elemental growth rate (REGR), reviewed by Peters and Bernstein (1997)]. In our simulations, we assumed that the REGR value is the same at all points of the curve, which implies that its length increases uniformly irrespective of the manner in which the curve is embedded in space.

**Fig. 4.**
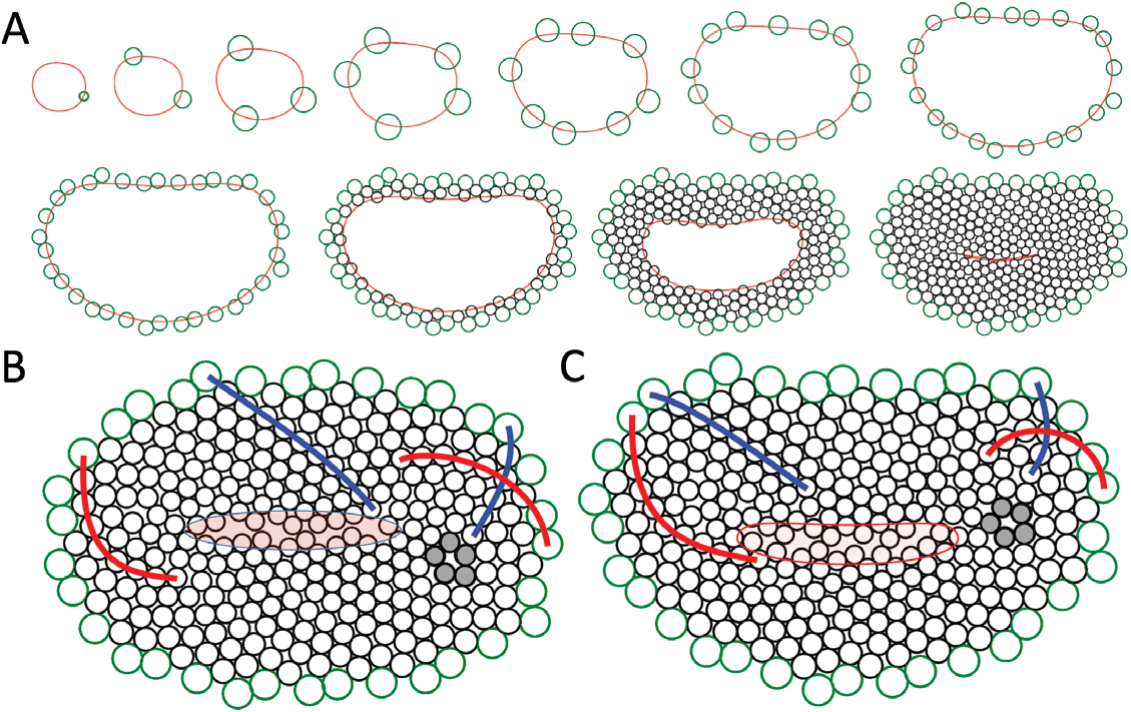
Simulated patterning of primordia in fasciated flower heads. (A) Snapshots of a simulation assuming the expansion and contraction of the active ring modeled as in Fig. 3B and C. Green circles represent primordia generated in Phases 1 and 2, black circles represent primordia generated in Phase 3. (B) Example of a pattern generated on an elliptic receptacle. (C) The final pattern produced by the developmental sequence (A). In (B) and (C), red and blue lines indicate sample parastichies forming families of 21 and 34 parastichies, respectively. The primordia shown in gray are an example of the pattern’s departure from regularity. The central part of each pattern, highlighted in transparent red, illustrates the gaps that emerge at the pattern closure. The models were implemented using the Virtual Laboratory 4.5.1 plant modeling software (algorithmicbotany.org/virtual_laboratory) running on MacBook Pro computers under macOS High Sierra v.10.13.6, and are available at algorithmicbotany.org/papers/fasciation2022.html.

The next question is how the availability of space for the insertion of new primordia should be measured (Schwabe, 1984). In the simulations we obtained the best results by defining the distance between two points as the length of the shortest path that connects them and lies entirely within the space between the head rim and the active ring (Fig. 3D). When the active ring coincides with the head rim, this distance is equivalent to the arc-length distance, measured along the rim curve; in the absence of obstacles and constraints, it amounts to the ‘usual’ straight-line Euclidean distance. As the exact computation of the path length is complicated (Hershberger *et al.*, 2013), we approximated it numerically. A new primordium is inserted at a location on the expanding and/or propagating active ring if the minimum distance between this location and the centers of the existing primordia exceeds a presumed threshold value. We observed that, in the presence of the lateral displacement of primordia described by Zhang *et al.* (2021), uniform intrinsic expansion of the active ring leads to an increase of primordia numbers in Phase 1 according to the Fibonacci sequence for a range of displacement rates irrespective of the rim shape (Fig. 4A; Supplementary Video S1). An additional displacement of primordia in the normal direction, simulating the early separation of the active ring from the head rim, leads to the emergence of the zig-zag pattern in Phase 2. The subsequent gradual shrinking of the ring leads to the filling of the head with primordia in Phase 3, with the zig-zag pattern providing a template for the emerging parastichies. Following the Fibonacci numbers of oppositely oriented ‘zigs’ and ‘zags’, parastichies tend to occur in Fibonacci numbers, as in the circular case, although the shape of parastichies varies according to the local curvature of the rim (Fig. 4B, C), as observed in fasciated heads under natural conditions (Szymanowska-Pułka, 1994). The generated patterns also exhibit local transitions, or departures from regularity (Fig. 4B, C), as observed in real phyllotactic patterns (Szpak and Zagórska-Marek, 2011; Golé *et al.*, 2016; Godin *et al.*, 2020). The proposed extension of the gerbera model (Zhang *et al.*, 2021) thus makes it possible to capture some essential features of phyllotactic patterns in fasciated heads.

**Fig. 5.**
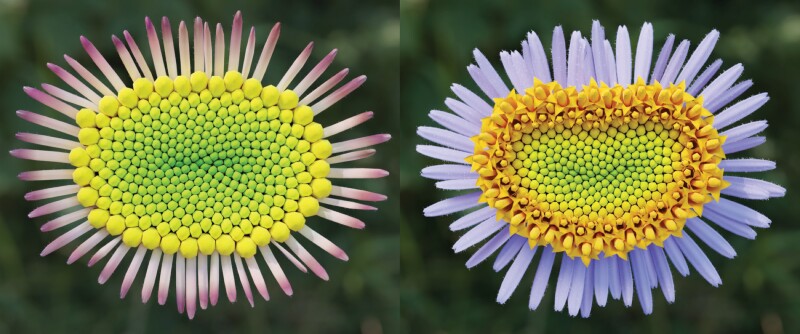
Rendered models of fleabane flower heads inspired by Fig. 1B and C. Positions of disk floret bases have been modeled as in Fig. 4B and C, but the florets were tilted, and the shapes of florets have been modified by collisions and mechanical interactions between their distal parts. These interactions masked the gaps between floret bases at the pattern closure, highlighted in transparent red in Fig. 4B and C. Ray florets were assumed to originate near the outermost disk florets. Individual florets were modeled using the interactive floret editor (Owens *et al.*, 2016) and Blender v2.81(https://blender.org), and incorporated into the model of phyllotaxis (Fig. 4B, C) with collisions between florets resolved as described by Owens *et al.* (2016). The final images were rendered using Blender.

We have also observed that the patterning process tends to produce gaps near the pattern closure (Fig. 4B, C)—a phenomenon also observed in circular heads (Fig. 1C, bottom), but exacerbated in linear closures. These gaps become better filled in the simulations that incorporate mechanical interactions between the distal parts of nearby florets, which push each other as they grow (Fig. 5; the interactions were simulated as described by Owens *et al.*, 2016). We hypothesize that similar interactions affect the appearance of flower heads in nature.

## Attributes of phyllotactic patterns in fasciated heads

**Fig. 6.**
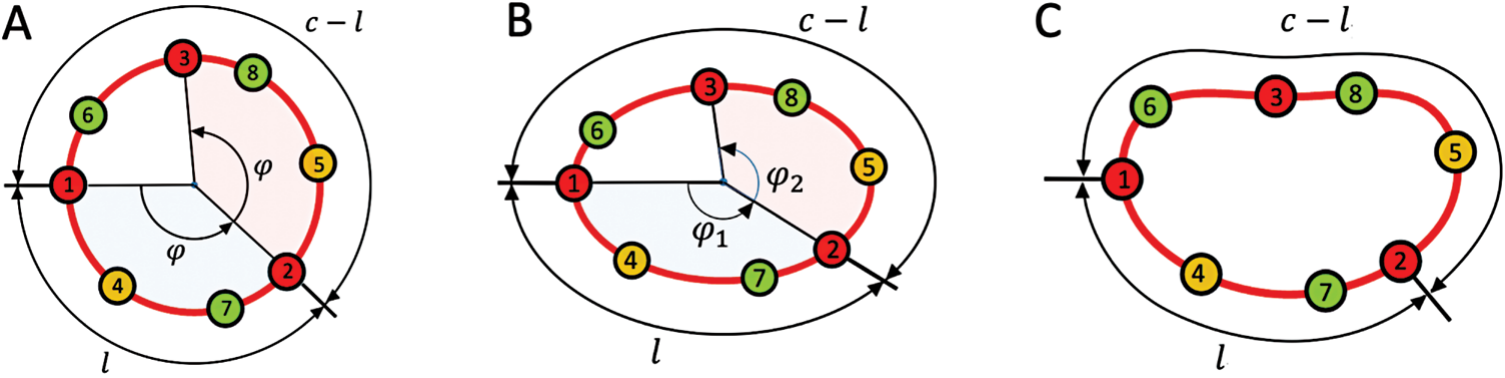
Extending the divergence angle to the divergence section. (A) In a circular head, primordia generated on the head rim can be arranged in a sequence with the divergence angle between consecutive primordia approximating the golden angle: φ≌137.5° (the equal angles between Primordia 1 and 2, and 2 and 3 are highlighted). This angle divides the full angle (360°) in the same ratio as the arc-length distance *l* between the primordia divides the total length *c* of the rim: $\frac{\;\varphi \;}{360{}^{\circ }-\;\varphi \;}=\frac{360{}^{\circ }-\;\varphi \;}{360{}^{\circ }}=\frac{1}{\tau }$ and $\frac{l}{c-l}=\frac{c-l}{c}=\frac{1}{\tau }$, where τ≌1.618 (the golden ratio). (B) In an elliptic head, consecutive primordia continue to divide the rim according to the golden ratio, but the divergence angles are no longer constant (φ_1_≠φ_2_) and thus, in general, are not equal to the golden angle. (C) In a more irregular head, consecutive primordia also divide the length of the rim according to the golden ratio, while the divergence angle cannot even be defined.

Several parameters have been developed over time to characterize and quantify phyllotactic patterning. Whether patterns in fasciated structures also exhibit such characteristics is thus an interesting question. In the context of *Brassica oleracea*, Kieffer *et al.* (1998) observed that ‘for “romanesco” it was impossible to carry out the biometrical analysis because branch apical meristems did not display any radial symmetry; however, the Fibonacci parastichy system could be determined from the number of contact parastichies.’ This observation highlights that some properties—in this case, the possibility of discerning parastichies and their tendency to appear in Fibonacci numbers—robustly carry over to fasciated heads, whereas other properties lose their significance. A conspicuous example is the divergence angle, defined as the angle between consecutively arising primordia, measured with respect to the head center. It has the remarkable property that, in circular heads, it often converges to the golden angle—approximately 137.5°—which results from the division of the full angle (360°) according to the golden ratio (Fig. 6A). Analyzing flowers of *D. winteri*, Doust (2001) noted that, in elliptic patterns, the divergence angle is no longer constant and fluctuates. In more irregular heads, which do not have a well-defined center, the divergence angle cannot even be meaningfully defined. Does this imply that the theory of phyllotaxis on fasciated heads is fundamentally impoverished compared with its circular counterpart?

Fortunately, this is not the case. We have already seen that the angular and radial distances measured with respect to the head center can be replaced with more general notions of arc-length and normal distances. This generalization makes it possible to express ring propagation and Hofmeister’s principle in the radially symmetric and fasciated heads alike. Following the same idea, we can generalize the divergence angle into the divergence section; that is, the ratio in which consecutive primordia divide the length of the active ring (Fig. 6B, C; see also Ryan *et al.*, 1991). Like the arc-length distance, the divergence section is an intrinsic geometric property of the pattern on a curve, meaning that it can be defined and measured irrespective of the curve shape. Moreover, especially in Phase 1, the interaction between existing primordia and the insertion of new primordia into the pattern is controlled by the arc-length distances. The resulting pattern properties are thus a function of these distances, not angles. The divergence angles are a derived notion, limited to the common, but special case of radially symmetric patterns. Their advantage is that they can be easily measured when the center of the pattern is identified, at least approximately.

In addition, the notions of divergence angle and divergence section put into focus the assumptions concerning the dynamics of primordia initiation. Since it is defined as the angle between consecutively arising primordia, irregularities in the order in which primordia are initiated affect the values of divergence angles (reviewed by Godin *et al.*, 2020). Such irregularities are particularly pronounced when parastichy numbers are high (Douady and Couder, 1996; Zhang *et al.*, 2021), and are exacerbated in fasciated heads. Nevertheless, it is often possible to disregard the precise order of primordia initiation (which, incidentally, may be difficult to identify experimentally), and order primordia at the head rim sequentially by assuming an approximately constant divergence section, close to the golden section, between consecutive primordia. This possibility suggests that, along with the parastichies, the divergence section may represent a robust characteristic of phyllotactic patterns, maintaining its significance irrespective of pattern symmetry and the dynamics of pattern formation.

## Conclusions and outlook

We extended a previously developed model of phyllotaxis in gerbera (Zhang *et al.*, 2021) to fasciated flower heads. A key to this extension was the observation that the gerbera model did not fundamentally depend on the assumption of circular symmetry. The extended model reproduced patterns of floret primordia observed in sample fasciated gerbera heads, as well as patterns of developed florets in fleabane (*Erigeron* sp.) heads (compare Figs 1 and 5). Specifically, it captured the arrangement of primordia and florets into conspicuous parastichies, the dependence of the parastichy shapes on the local curvature of the head rim, and the tendency of parastichies to occur in Fibonacci numbers in spite of the fasciation (Fig. 4). These features support the model, but also highlight the need for its further validation—one of the numerous challenging research problems posed by fasciation and patterning of fasciated meristems.

The open problems cross the boundaries of biology and mathematics. From a biological perspective, key questions include the regulation and dynamics of the fasciated head growth and active ring propagation. *In vivo* imaging of developing heads over an extended period of time, from the patterning of the first primordia in Phase 1 to the pattern closure at the end of Phase 3, is outside the capabilities of current experimental methods. The dynamics of head growth and phyllotactic patterning in non-fasciated gerbera heads were inferred from observations of different heads at different stages of development, as the current methods allow live-imaging of single heads only for a limited time (Zhang *et al.*, 2021). For fasciated heads a similar approach is complicated by the diversity of head shapes and the relative rarity of the occurrence of fasciation. The study of mutants in which fasciation is common, such as the *GhCLV3* mutants in gerbera, may alleviate the latter concern.

Mutants may also be helpful in studying the relationship between fasciation and meristem/receptacle size. As we noted earlier, the enlargement of meristems is often associated with fasciation; however, the causal nature of this link remains unclear. An explanation may answer the question of the origins of fasciation and, perhaps more importantly, elucidate the mechanisms that foster the approximately circular shape of ‘normal’ meristems.

The gerbera model and its fasciated extension are based on the Hofmeister/Snow and Snow hypothesis, according to which new primordia emerge when and where there is enough space for them. While intuitively clear, this criterion does not specify how the availability of space is measured. In the simulations shown here, we employed path length distance. It would be interesting to analyze, through molecular-level observations of all phases of patterning, if this distance indeed reflects the paths of information flow in developing flower heads and, more generally, properly captures the biological reality of measuring the availability of space. It would also be worthwhile to confirm through direct observation of auxin maxima that the essential element of Phase 1—the lateral displacement of primordia towards their older neighbors—occurs in fasciated heads as it does in circular heads (Zhang *et al.*, 2021).

Of additional interest is the pattern closure at the end of Phase 3. In circular heads, this closure is manifested by the irregular placement of primordia in the small zone near the head center. In fasciated heads, closures are often linear. Examples of flowers with multiple carpels suggest that linear closures may even be branched (Endress, 2014). These closures create new types of neighborhood relationships, absent in the typical development of circular heads, and thus may provide additional information regarding the distance measures and positioning mechanisms in play. The underlying question is: how are the linear closures filled? Are the primordia themselves displaced to better fill the gaps? This possibility is of conceptual interest, as it could relate the observations of primordia displacement in radial (Galvan-Ampudia *et al.*, 2020) and lateral (Zhang *et al.*, 2021) directions to the contact pressure theory of phyllotaxis (Schwendener, 1878; Adler, 1974; Ridley, 1982), which postulates displacement of primordia as a means of obtaining better packing. Another (not mutually exclusive) possibility is that florets are attached to the receptacle in a pattern with gaps, but these gaps are masked by the subsequent tilting and bending of florets due to the mechanical interactions between their distal parts as they grow. We employed the latter technique in the simulations shown in Fig. 5, but the question of how the gaps are filled or masked in nature requires further detailed analysis of the developing heads.

From a mathematical point of view, fasciated heads suggest a re-examination of the fundamental notions used to describe and quantify phyllotactic patterns. It appears that they fall into two categories. (i) Some notions preserve their definitions and meaning in fasciated patterns. Examples include parastichies and meristic characteristics, that is the numbers of organs, such as bracts or ray florets, which are often related to the number of parastichies or florets on the rim (Battjes and Prusinkiewicz, 1998, and references therein). (ii) Other notions lose their significance or cannot be defined in fasciated structures. These are, above all, the notions that refer to the head center: the divergence angle, the distances of primordia measured from the pattern center, and the plastochron ratio, which is built on the basis of these distances (Jean, 1994; Barabé and Lacroix, 2020; Godin *et al.*, 2020). Some of these notions can regain their relevance using an appropriately generalized definition, as we have shown by generalizing the divergence angle to the divergence section as an example.

In conclusion, studies of fasciated heads help focus attention on the essential properties of phyllotactic patterning, stemming directly from the mechanism of patterning captured by the Hofmeister/Snow and Snow hypothesis, while distinguishing them from the properties founded on additional assumptions, such as the circularity of the head. Despite centuries of research, phyllotaxis continues to generate fascinating research problems at the intersection of biology, mathematics, and computer science.

## Supplementary data

The following supplementary data are available at *JXB* online.

Video S1. Simulation of phyllotactic patterning on a fasciated head.

erac101_suppl_Supplementary_Video_S1

## Data Availability

The models used to generate phyllotactic patterns on fasciated heads are available at algorithmicbotany.org/papers/fasciation2022.html. Their execution requires the Virtual Laboratory plant modeling software available at algorithmicbotany.org/virtual_laboratory.
